# Development and validation of an AI-enabled digital breast cancer assay to predict early-stage breast cancer recurrence within 6 years

**DOI:** 10.1186/s13058-022-01592-2

**Published:** 2022-12-20

**Authors:** Gerardo Fernandez, Marcel Prastawa, Abishek Sainath Madduri, Richard Scott, Bahram Marami, Nina Shpalensky, Krystal Cascetta, Mary Sawyer, Monica Chan, Giovanni Koll, Alexander Shtabsky, Aaron Feliz, Thomas Hansen, Brandon Veremis, Carlos Cordon-Cardo, Jack Zeineh, Michael J. Donovan

**Affiliations:** 1 PreciseDx, 1111 Amsterdam, Stuyvesant Building 8-822, New York, NY 10025 USA; 2 Icahn School of Medicine at Mount Sinai, New York, NY USA; 3 Mount Sinai Hospital, New York, NY USA; 4 Department of Pathology, University of Miami, Miami, FL USA

**Keywords:** Breast cancer, Prognostic grade, Artificial intelligent image analysis

## Abstract

**Background:**

Breast cancer (BC) grading plays a critical role in patient management despite the considerable inter- and intra-observer variability, highlighting the need for decision support tools to improve reproducibility and prognostic accuracy for use in clinical practice. The objective was to evaluate the ability of a digital artificial intelligence (AI) assay (PDxBr) to enrich BC grading and improve risk categorization for predicting recurrence.

**Methods:**

In our population-based longitudinal clinical development and validation study, we enrolled 2075 patients from Mount Sinai Hospital with infiltrating ductal carcinoma of the breast. With 3:1 balanced training and validation cohorts, patients were retrospectively followed for a median of 6 years. The main outcome was to validate an automated BC phenotyping system combined with clinical features to produce a binomial risk score predicting BC recurrence at diagnosis.

**Results:**

The PDxBr training model (*n* = 1559 patients) had a C-index of 0.78 (95% CI, 0.76–0.81) versus clinical 0.71 (95% CI, 0.67–0.74) and image feature models 0.72 (95% CI, 0.70–0.74). A risk score of 58 (scale 0–100) stratified patients as low or high risk, hazard ratio (HR) 5.5 (95% CI 4.19–7.2, *p* < 0.001), with a sensitivity 0.71, specificity 0.77, NPV 0.95, and PPV 0.32 for predicting BC recurrence within 6 years. In the validation cohort (*n* = 516), the C-index was 0.75 (95% CI, 0.72–0.79) versus clinical 0.71 (95% CI 0.66–0.75) versus image feature models 0.67 (95% CI, 0.63–071). The validation cohort had an HR of 4.4 (95% CI 2.7–7.1, *p* < 0.001), sensitivity of 0.60, specificity 0.77, NPV 0.94, and PPV 0.24 for predicting BC recurrence within 6 years. PDxBr also improved Oncotype Recurrence Score (RS) performance: RS 31 cutoff, C-index of 0.36 (95% CI 0.26–0.45), sensitivity 37%, specificity 48%, HR 0.48, *p* = 0.04 versus Oncotype RS plus AI-grade C-index 0.72 (95% CI 0.67–0.79), sensitivity 78%, specificity 49%, HR 4.6, *p* < 0.001 versus Oncotype RS plus PDxBr, C-index 0.76 (95% CI 0.70–0.82), sensitivity 67%, specificity 80%, HR 6.1, *p* < 0.001.

**Conclusions:**

PDxBr is a digital BC test combining automated AI-BC prognostic grade with clinical–pathologic features to predict the risk of early-stage BC recurrence. With future validation studies, we anticipate the PDxBr model will enrich current gene expression assays and enhance treatment decision-making.

**Supplementary Information:**

The online version contains supplementary material available at 10.1186/s13058-022-01592-2.

## Background

Histopathologic characterization of all solid tumors is a critical first step in diagnostic classification (i.e., organ/cell type) and development of the histologic grade or state of differentiation. Surgical pathologists have used the term differentiation, or histologic grade, as a prognostic feature to communicate tumor aggressiveness and the likelihood of spread with increasing risk [1, 2]. The challenge with all tumor grading systems, regardless of the tumor type, is that the scoring is subjective, interpretive, semiquantitative, skill-dependent, and oftentimes inconsistent and variable [3]. These deficiencies are most pronounced in tumor types that rely on complex decision-based scoring systems, which are important components in establishing clinical risk, such as those for both prostate and breast cancer (BC) [4].

We focused on invasive BC, which is histologically graded using the Nottingham grading system (NGS) consisting of three features: tubule structures (gland architecture), nuclear pleomorphism (nucleus size and shape), and the number of mitotically active cells within a pre-defined field of view. The challenge is that NGS has a reported 25–30% intra- and inter-pathologist discordance, especially in the moderately differentiated (grade 2) category. Despite the reported misclassification and lack of standardization, grade continues to play an important prognostic role in patient management from neoadjuvant therapy choice to implementation of genetic testing results [4–7].

The most recent guidelines from national organizations [8–10] emphasize the importance of a complete pathology assessment of invasive BC (i.e., tumor size, grade, endocrine receptor status, and HER2 amplification) prior to genomic test selection and therapeutic sequencing, including surgery. In addition, as approximately 50% of pathology reports are missing elements critical to patient management, specifically grade and margin status, consistency in pathology reporting is required. Although not robust, mitotic figure activity, which reflects proliferation, is one of the most important variables to predict outcome but also the most common cause of discordance due to staining artifacts, mimickers, and tumor cellularity [3, 9]. Finally, analysis of molecular signatures revealed that BC grade remains an independent risk factor in multivariate models and provides additional information to improve BC subtyping beyond endocrine status and HER2 overexpression [11, 12]. This is clinically significant, as BC grade is an independent prognostic feature and plays a direct role in whether patients are managed via neoadjuvant therapy or surgery, can affect psychological well-being, and assists in the interpretation of genomic-based risk assessments such as MammaPrint, EndoPredict, and Oncotype DX Breast Recurrence Scores [12].

Here, we developed a deep learning system for analyzing invasive BC histology images with the purpose of enhancing and improving the current BC grading approach. We created an AI-digital BC grade and incorporated relevant clinical data to produce a BC recurrence risk test [13, 14]. Our primary objective was to rely only on the BC H&E digital image and readily available clinical data to both standardize BC grading and provide an accessible tool to predict breast cancer recurrence within 6 years.

## Methods

### Study design

We performed a retrospective longitudinal clinical development and validation study utilizing samples from breast cancer patients within the Mount Sinai Health Care System (which included Mount Sinai Hospital, and Mount Sinai Beth Israel, NYC, NY) from 2004 to 2016. Eligible participants were ≥ 23 years old with infiltrating ductal or mixed ductal and lobular carcinoma of the breast (IDC) and a median 6-year follow-up data available. Patients treated with neoadjuvant therapy or prior history of BC were excluded. The institutional review board approved the use of human patient specimens and their clinical data for this study and waived informed consent. This study adhered to the TRIPOD checklist guidelines to ensure transparency of the reporting of our prediction model study.

All participants had H&E slides available for analysis (Department of Pathology, Mount Sinai Hospital, NYC, NY) or paraffin blocks for slide generation that had been procured from prior resected breast cancer specimen investigation. H&E slides were digitized (40X magnification) using a Philips UltraFast Digital slide scanner (Netherlands). A total of 15,000 H&E slides and paired digital images (40X magnification) were reviewed with single whole slide images (WSI) selected for model development. The manual histologic grade was obtained from the original pathology report. Two pathologists (AS, BV), blinded to outcome, reviewed all cases to confirm the diagnosis of invasive breast cancer and tumor/image quality; no subjects were rejected. One image per patient was advanced for feature extraction and model development. Clinical and pathology data were extracted from the Mount Sinai electronic medical record system (EPIC). The data were stored in a Department of Pathology secure Web-based proprietary software platform for cohort construction and statistical analysis.

### Image feature construction/digital image analysis

**Fig. 1:**
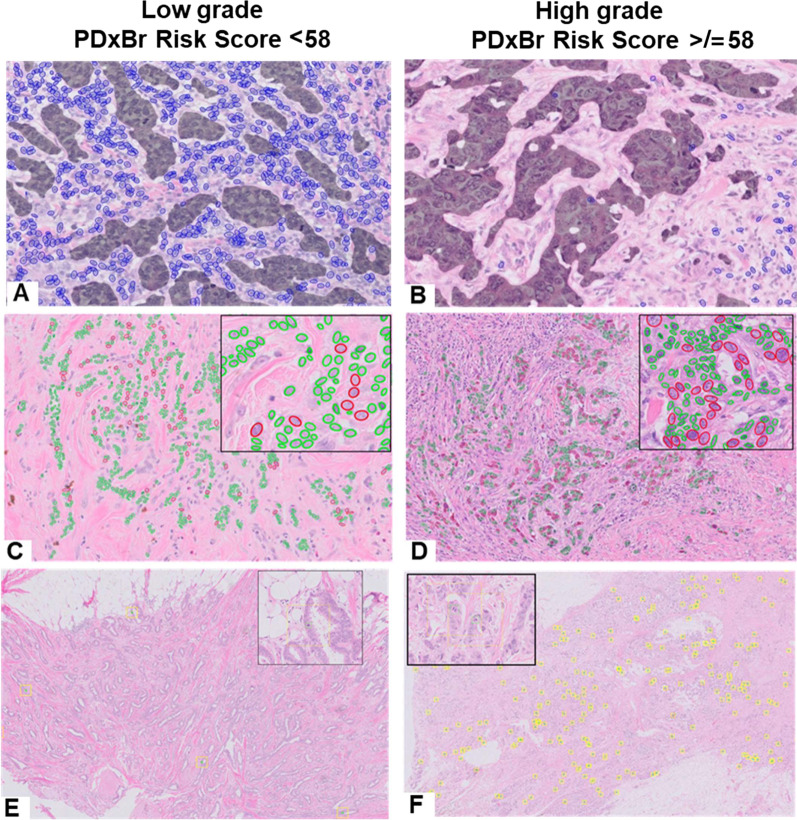
PDxBr AI-Digital Grade Features. Representative images of **A**–**B** tumor-infiltrating lymphocyte feature that detects and quantitates lymphocytes (highlighted in blue) in the periepithelial area of invasive tumor (dark overlay); **C**-**D** nuclear pleomorphism feature that quantitates the difference between the largest epithelial nuclei (outlined in red) and the average nuclei (outlined in green) in the invasive tumor; and **E**–**F** mitotic count feature, which uses clustering methods to identify the regions of highest proliferation (yellow outline—equivalent to 10 high-power fields)

**Fig. 2:**
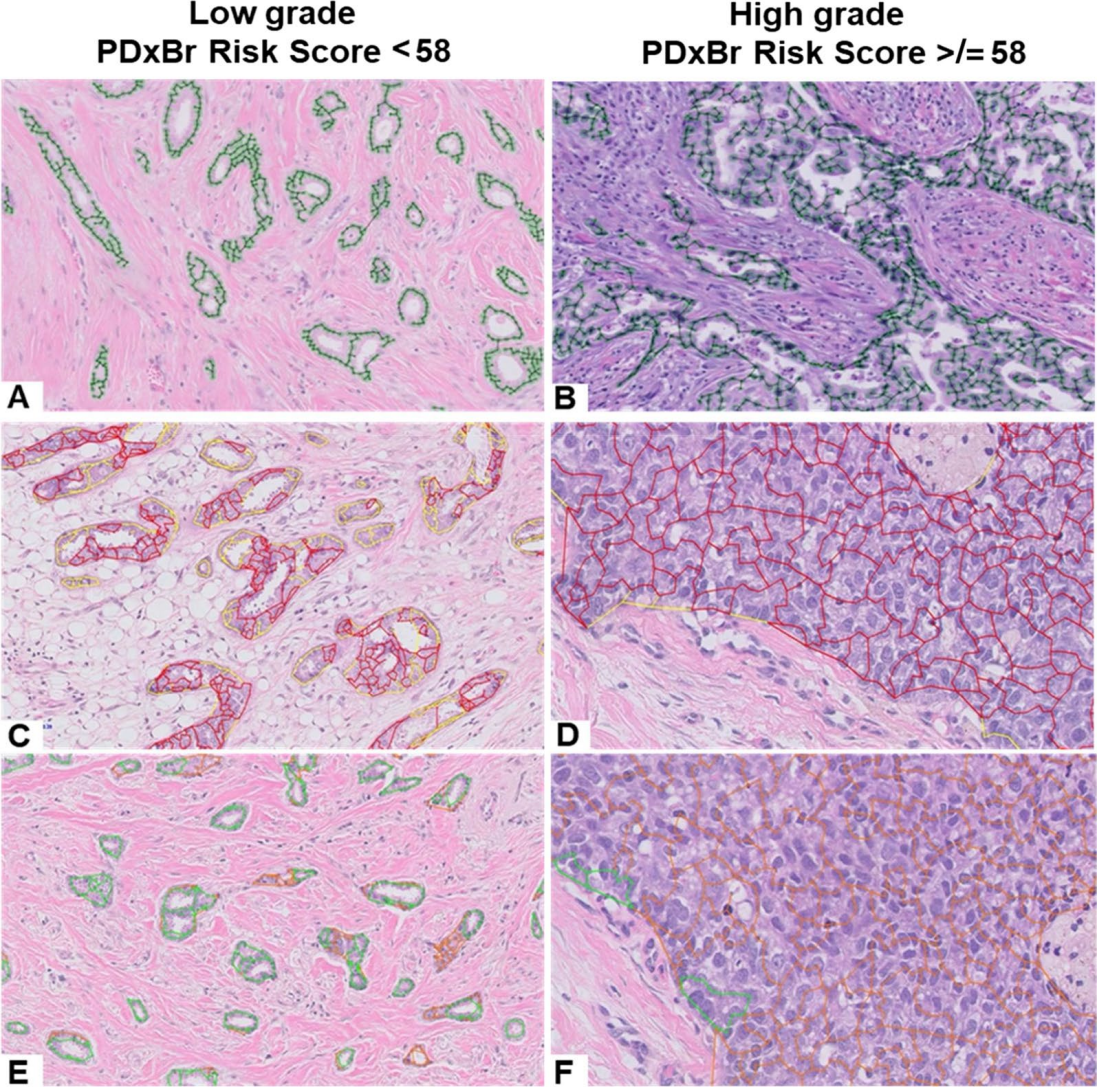
PDxBr AI-Digital Grade Architectural Features. Representative patient images of **A**–**B** the graph-based approach to establish the extent of tubule formation in invasive epithelial structures by quantifying the degree of graph branching; **C**–**D** quantitates the degree the invasive epithelium is growing in sheets, high feature value shown in red and low feature value in yellow; **E**–**F** quantitates the degree the invasive epithelium is growing in tubular structures, high feature value shown in green and low feature value in orange

Approved WSI were interrogated with a deep learning morphology feature array (MFA) to extract tumor cell and tissue architectural features [15] (Figs. 1 and 2) to produce individual cell and tissue BC features which were prioritized based on the risk of BC recurrence with a concordance index (c-index) range of < 0.4 or > 0.4. The resultant 800-curated features represented BC grading and tumor composition. After outcome filtering and comprehensive mini-performance models, 40 features passed for model development. (Supplemental details are provided in Additional file 1.)

### Primary objective and study endpoints

The primary objective was to assess the performance and accuracy of a novel, AI-digital BC grade and clinical feature test to predict the likelihood of BC recurrence within 6 years. The composite BC recurrence study endpoints included: (1) disease-free survival, time from diagnosis to first event including ipsilateral BC recurrence, local recurrence, regional recurrence, distant recurrence, contralateral second primary, or death without evidence of recurrence; (2) distant recurrence-free interval, time from diagnosis to date of distant recurrence or death with distant recurrence; (3) relapse-free interval, time from diagnosis to first recurrence (e.g., ipsilateral, loco-regional, distant, metastatic) or date of death with recurrence; and (4) overall survival, time from diagnosis to death of any cause.

### Statistical analysis

Demographic and event-balanced training and validation cohorts (2004–2016) were used for model development with the c-index/area under the curve (AUC) and Kaplan–Meier survival analyses. C-indices and hazard ratios (HR) are reported as 95% CIs. Significance was set as a two-tailed *p* < 0.05. The only clinical variables selected by the model included age, tumor size, American Joint Committee on Cancer (AJCC) v8 stage, and lymph node status while pathologist grade, estrogen and progesterone receptor status, and HER2 amplification were available but not selected. The training performance of clinical and imaging features was modeled (unadjusted) through the construction of a support vector regression analysis with censored data (SVRc) [16].

Features in the model are covariates that employ a linear kernel where the hyperparameters are optimized through particle swarm optimization [17], allowing for both censored and uncensored events to be appropriately weighted during threshold application. An SVRc threshold, which maximizes sensitivity and specificity, is selected by the algorithm that divides the dataset into high-risk and low-risk categories. Measures of predictive accuracy include negative predictive value (NPV), positive predictive value (PPV), sensitivity, and specificity. The output is a risk score from 0 to 100 representing an individual’s risk of experiencing an event, with higher numbers indicating increased risk.

A subgroup analysis was also performed utilizing only those patients with an observed Oncotype Recurrence Score (RS, scale of 0–100) compared with our newly developed PDxBR risk score based on AUC, NPV, and PPV.

## Results

### Patient and tumor characteristics

A total of 2075 eligible participants were subdivided (3:1) into training (*n* = 1559) and validation (*n* = 516) cohorts. Patient characteristics were similar overall (Table 1). The majority of samples were estrogen receptor (ER, 87%) and progesterone receptor (PR, 81%) positive, (i.e., Luminal A) with 12% (*n* = 252) Her2 amplified, and 8% (*n* = 160) triple negative. 42% and 40% histologic grade 2 and 3, respectively. Given the absence of Ki67 and only 2% of low-level ER + cases, the luminal B categorization was not feasible. Approximately 70% received tamoxifen and 74% had chemotherapy in addition to endocrine or radiation treatment. There were 289 (14%) total recurrence events (220 in training and 69 in validation) including metastases (*n* = 85), loco-regional and nodal extension (*n* = 72), and overall survival (*n* = 126). All available clinical features (including pathologist histology grade) were included for model development. There was no imputation necessary for any clinical variables.

**Table 1:** Demographics of the PDxBr training and validation cohorts

	Train (*N* = 1559)	Validation (*N* = 516)
Median age, y (range)	60 (24, 90)	60 (28, 90)
Race/ethnicity, *N* (%)		
Asian	9 (0.6)	6 (1)
Black	81 (5)	21 (4)
Latino	22 (1)	8 (1)
Other	186 (12)	69 (13)
Unknown	408 (26)	128 (25)
White	853 (55)	284 (55)
Estrogen receptor, *N* (%)		
0	204 (13)	68 (13)
1	1355 (87)	448 (87)
Progesterone receptor, *N* (%)		
0	292 (19)	100 (19)
1	1267 (81)	416 (81)
HER2, *N* (%)		
0	1362 (87)	461 (89)
1	197 (13)	55 (11)
Tumor size (cm)	1.5 ± 1.1 (0.1, 17.0)	1.5 ± 0.9 [0.1, 8.0]
T1	1168 (75)	398 (77)
T2	367 (24)	112 (22)
T3	24 (2)	6 (1)
Stage		
Stage1	1055 (68)	362 (70)
Stage 2	386 (25)	123 (24)
Stage IIIA/B	81 (5)	21 (4)
Stage IIC	36 (2)	10 (2)
Stage IV	1 (0.1)	0 (0)
Lymph node status		
posLN = 0	1075 (69)	350 (68)
microLN or isolatedLN and posLN = 0	127 (8)	56 (11)
1 ≤ posLN ≤ 3	239 (15)	79 (15)
posLN > 3	118 (8)	31 (6)
Grade		
1	290 (19)	68 (13)
2	649 (42)	219 (42)
3	620 (40)	229 (44)
Total events		
0	1339 (86)	447 (87)
1	220 (14)	69 (13)
Time to event (months)	75.3 [-16.0, 68.0, 200.0]	79.4 [0.0, 69.0, 1173.0]

*LN* Lymph node

### PDxBr—training

**Fig. 3:**
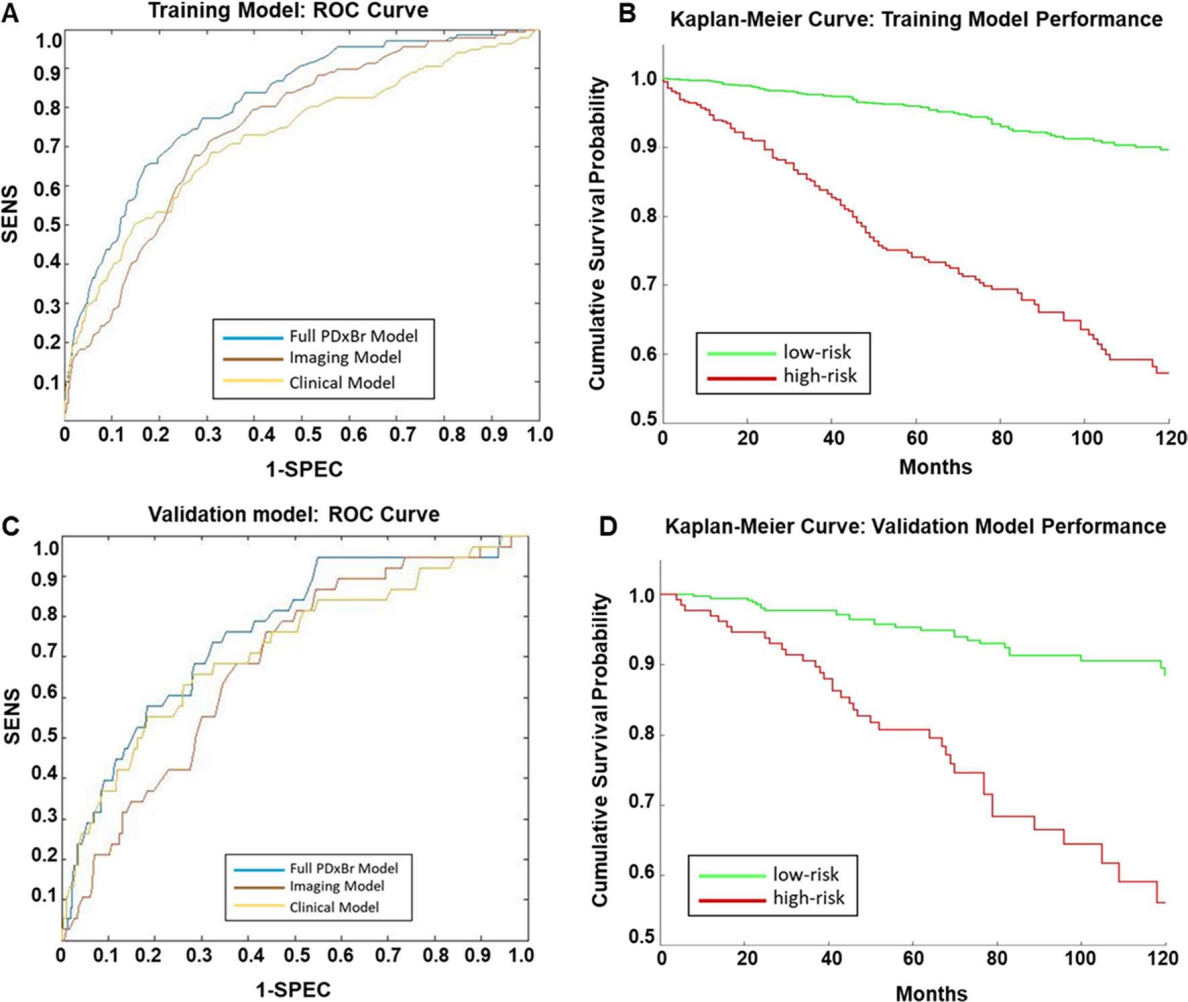
Accuracy of training and validation models predicted recurrence risk and performance in patients stratified by high and low risk of recurrence. AUC-ROC curves of **A** training model and **C** validation model compared with the optimized clinical model and image feature model. Kaplan–Meier curve of **B** training and **D** validation model performance with a cutoff distributing patients as high or low risk for breast cancer recurrence

The PDxBr training model (*n* = 1559) consisted of the following clinical variables: age, age combined with tumor size (cm), constructed to balance the importance of tumor size in conjunction with age, anatomic stage, and lymph node (LN) status as well as 7-imaging features (Table 2), differentially weighted through SVRc to identify patients at increased risk of early BC recurrence within 6 years. Representative images of PDxBr model grade features are displayed in Fig. 1 and representative images of PDxBr model architectural features are displayed in Fig. 2. The two most important clinical variables included were positive lymph nodes and age at diagnosis, while the two most significant image features represent tubule formation (degree of differentiation) and tumor-adjacent lymphoid clusters (< lymphocytes in the tumor = greater chance of recurrence). The value and direction (positive or negative) of each feature equates to its weight within the model and impact on outcome. Neither histology grade nor ER/PR/Her2 status was selected by the model. As shown in Fig. 3A, the PDxBr assay was superior to either an optimized clinical-only model (consisting of age, tumor size, stage and lymph node positivity) or an AI imaging feature model for predicting BC recurrence (*p* < 0.001, Additional file 2: Table S1 and Additional file 3: Table S2). The training AUC/C-index of the PDxBr was 0.78 (95% CI, 0.76–0.80) versus optimized clinical model 0.71 (95% CI, 0.67–0.74) and AI imaging of 0.72 (95% CI, 0.70–0.74, *p* < 0.001). The AI imaging model produced a binomial low- and high-risk classification, resulting in the redistribution of the NGS grade 2 into low- versus high-risk categories (see the following section). Finally, since Adjuvant! is no longer available we also evaluated the performance of PDxBr with the binary clinical risk categorization algorithm used in the MINDACT trial [18, 19]. Of note, the clinical risk algorithm is very similar to the PDxBr clinical algorithm, except that age is not included and histologic grade is utilized. C-index/AUC for clinical risk was 0.63 (95% CI 0.6–0.65) versus PDxBr of 0.78 (95%CI 0.76–0.8), demonstrating a significant improvement (see Additional File 4: Fig. S1). The demographics of the clinical high-risk group were as expected including 19% Her2 +ve, 70% NGS grade 3, 29% 1–3 positive LN and > 2 cm.

**Table 2:** PDxBr training and validation: image and clinical features

Training dataset	
Concordance index (C-index):	0.78
*Training sensitivity/specificity threshold:	57.77
Train sensitivity:	0.72
Train specificity:	0.77
Validation dataset	
Concordance index (C-index):	0.75
Test sensitivity:	0.61
Test specificity:	0.77

Feature	Weight in final model
Proliferative activity	− 17.11
Nuclear pleomorphism	− 28.53
Age and size composite	− 11.07
Age at diagnosis	− 23.14
Stage	− 12.21
Tumor-infiltrating lymphocytes	23.48
Positive lymph nodes	− 29.34
Tumor sheets/architecture	8.36
Intact tubules	42.91

Image features: Proliferative activity: mitotic figure count; nuclear pleomorphism: nuclear shape, size, contour, chromatin content; tumor-infiltrating lymphocytes: number of intra-tumoral lymphocytes; tumor sheets/architecture: concentrated islands of tumor with and without intervening stroma; intact tubules: varying sized gland structures composed of epithelial cells with an intact lumen and adjacent stromal components. Clinical feature: age and size composite; novel feature that balances impact of tumor size as a function of age

*Model threshold of 57.77 was rounded to 58 for subsequent risk categorization and reporting

Subjects stratified by the PDxBr risk score below (< 58, low risk) versus above (high risk, ≥ 58) yielded an HR of 5.5 (95% CI 4.2–7.2), *p* < 0.001 with sensitivity 0.72, specificity 0.77, NPV 0.95, and PPV 0.32 for predicting BC recurrence (Table 3 and Fig. 3B). Increasing risk scores indicated a greater risk of recurrence within 6 years of which 72% were low risk and 28% high. Of the 220 events in training, there were 22 in the triple negative (TN) and 30 in the Her2 + subgroups for which the model classified 17/22 (77%) and 20/30 (67%) as high risk, respectively.

**Table 3:** Utilization of the PDxBr training and validation models with cutoff to stratify patients into high- and low-risk recurrence groups

	Events	Censored	Total
Training model			
*Risk score ≥ 58	140	295	435
*Risk score < 58	80	1044	1124
Training performance metric		Performance (95% CI)	
Sensitivity		0.715	
Specificity		0.772	
PPV		0.319	
NPV		0.948	
Confidence interval		0.787 (0.764, 0.808)	
Validation model			
Risk score ≥ 58	39	104	143
Risk score < 58	30	343	373
Performance metric		Performance (95% CI)	
Sensitivity		0.605	
Specificity		0.768	
PPV		0.242	
NPV		0.941	
Confidence interval		0.755 (0.717, 0.792)	

*PPV* positive predictive value, *NPV* negative predictive value

*Model cutoff of 57.77 rounded to 58

### PDxBr—validation

In validation, the AUC/C-index of the PDxBr (*n* = 516) improved risk discrimination (0.75, 95% CI, 0.72–0.79) when compared with either the clinical (0.71, 95% CI, 0.66–0.75) or image (0.67, CI, 0.63–0.71, *p* < 0.001 for both) features only models (Fig. 3C and Table 2 and Additional file 2: Table S1 and Additional file 3: Table S2). By comparison, C-index/AUC for the binary clinical risk in the validation cohort was 0.64 (95% CI 0.6–0.68) (see Additional File 4: Fig. S1B). When patients were stratified by the PDxBr risk score of 58, the HR was 4.4 (95% CI 2.7–7.1, *p* < 0.001), sensitivity 0.60, specificity 0.77, NPV 0.94, and PPV of 0.24 for predicting BC recurrence (Table 3 and Fig. 3D). Comparable to training, 72% were classified as low risk and 28% high. Of the 69 events in validation, there were 10 in the triple negative (TN) and 8 in the Her2 + subgroups for which the model classified 6 (60%) and 7 (87%) as high risk, respectively. The demographics of the clinical high-risk group in the validation cohort were identical to the clinical high risk in training.

### PDxBr AI grade versus pathologist-assigned breast cancer grade

We compared the pathologist histologic grade with the PDxBr test and the AI grade (image-only model) utilizing the existing training and validation cohorts. Of note, the pathologist histologic grade as an independent clinical feature was not selected during model development. When the full PDxBr model is evaluated with AUC/C-index in training (Tr) and validation (Val) the significant incremental improvement for predicting BC recurrence is primarily driven by the univariate performance of AI grade (CI: Tr 0.72, Val 0.68) when compared to histologic grade (CI: Tr, 0.64, Val, 0.61) versus final PDxBr model (CI: Tr 0.78, Val, 0.75, *p* < 0.001). Kaplan–Meier curves comparing AI grade versus histology grade reflect significant differences in both training (HR: 3.65, *p *value < 0.001) and validation models (HR: 2.1, *p* < 0.001, Additional file 5: Fig. S2).

With the PDXBr model, 187 (22%) of the 868 NGS grade 2 patients (representing 41% of 2075) were reclassified as high risk and 681 (78%) as low risk. With AI grade, 177 (20%) were reclassified as high risk and 691 (80%) as low risk, suggesting that the NGS grade 2 is a hybrid of NGS grade of 1 and 3.

For the 358 NGS grade 1 cases (17% of 2075), PDxBr reclassified 15 (4%) as high risk and 343 (96%) as low risk, while the AI grade reclassified 5 (1%) as high risk and confirmed 353 (99%) as low risk. Importantly, for the 849 NGS grade 3 patients (41% of 2075), the PDxBr confirmed 377 (44%) as high risk and reclassified 506 (66%) as low risk, while AI grade confirmed 115 (14%) as high risk and 734 (86%) as low risk. This adjustment in grade characterization will impact the performance of clinical risk models (e.g., MindAct), which in this cohort was non-contributory for predicting recurrence with CI’s of 0.63 in training and 0.64 in test. (Additional file 4: Fig. S1).

### PDxBr improves oncotype recurrence score (RS) risk discrimination: subgroup analysis

We also evaluated the performance of the PDxBr compared to the gene expression assay (Oncotype DX) Breast in a subpopulation (*n* = 599), combining the training and validation groups to optimize events (*n* = 36, 6%). Of the 36 events, 21 (60%) were local–regional recurrences. Cohort characteristics were similar to the larger cohort (Additional File 6: Table S3). Of note, there were 55% histologic Grade 2 cases in this population. Combining Oncotype model with assorted sub-models including histologic grade, clinical features, AI grade, or PDxBr model (see Additional File 7 : Tables S4A–D, for complete model characteristics) in a SVRc analysis demonstrated incremental improvement in the C-index for predicting BC recurrence (Table 4 and Additional File 8: Fig. S3): Oncotype RS: Ci 0.63 (95% CI 0.55–0.71) versus Oncotype RS and AI grade, Ci 0.73 (95% CI 0.65–0.80) versus Oncotype RS and PDxBr, Ci 0.76 (95%CI 0.70–0.82). Applying a cutoff to identify low- versus high-risk patients showed improvement over oncotype alone: Oncotype RS: sensitivity 42%, specificity 86%, HR 3.5 *p* < 0.01 versus Oncotype RS and AI image features: sensitivity 72%, specificity 82%, HR 6.1 *p* < 0.001 versus Oncotype RS and PDxBr: sensitivity 66%, specificity 80%, HR 6.1, *p* < 0.001.

**Table 4:** AUC comparison of oncotype alone and then combined with histology grade, clinical data (age, stage, tumor size, and LN pos), AI grade, and the PDxBr model

Model	C-index	CI lower limit	CI upper limit
Oncotype RS	0.35	0.26	0.48
Oncotype + grade	0.51	0.42	0.60
Oncotype + Clinical	0.64	0.57	0.71
Oncotype + AI grade	0.72	0.67	0.79
Oncotype + PDX Br	0.76	0.70	0.82

In the oncotype RS plus PDxBr model, there were 14 events in the low-risk population: local regional recurrences (*n* = 10), metastasis (*n* = 2), and death (*n* = 2). By comparison, in the oncotype RS-only low-risk (≤ 25) group there were 24 events: local regional recurrences (*n* = 16), metastasis (*n* = 5), and death (*n* = 3).

## Discussion

We developed a novel AI-enabled digital platform to identify and phenotype infiltrating ductal (with mixed lobular/ductal) breast carcinoma from an H&E-stained image. By applying image analysis tools to isolate and quantify individual elements of the invasive cancer, we extracted features representing tissue architecture and cell type characteristics, which we call the AI grade. This platform identifies discrete biologically driven tumor elements that are not captured using current breast cancer grading such as the NGS [14]. In contrast to other approaches in computational histopathology that focus on classification problems (e.g., tumor versus no tumor) with multi-instance learning [20], we employ a supervised pathologist driven feature design process. The result is readily explainable features for pathologists and oncologists, appropriate for risk modeling and potential predictive response. We then combined the AI grade with clinical features such as the patients’ age, tumor size, stage and number of positive lymph nodes to generate the PDxBr risk score to categorize patients as low or high risk for BC recurrence within 6 years of a definitive treatment (i.e., lumpectomy or mastectomy). The initial objective was to develop a readily accessible tool that had utility for patients with early-stage ER + invasive BC. However, we also observed that the PDxBr model was able to effectively risk stratify patients with Her2 +ve and triple-negative disease by identifying 50 of the combined 70 events (70%), relying only on age, tumor size, stage, lymph node status and their H&E AI-grade phenotype.

Validation of the PDxBr model produced a C-index of 0.75 versus optimized clinical or imaging models of 0.71 and 0.67, respectively. Using a validated risk score to discriminate low versus high risk yielded an HR of 4.4 (*p* < 0.001) with an NPV of 94. The data suggest that with an NPV of 94% and a PPV of 24%, due to the lower prevalence of events in this low-risk cohort, the PDxBr would potentially be effective in combination with oncotype RS and other gene expression tests to rule-out chemotherapy. Moreover, our studies comparing histologic grade versus AI grade provide evidence of improved risk discrimination and the importance of advancing objective and adaptive (biological intent) grading systems for invasive BC. In the PDxBr model, histology grade (generated by experienced BC pathologists) has been replaced by the AI features and additional tumor-infiltrating lymphocyte feature provides a prognostic/predictive role for immune characterization in the pre-treatment setting. Additional studies are underway to characterize the spatial and organizational properties of these infiltrates and their relationship with invasive cancer and recurrence.

The seminal paper promoting the use of gene expression to predict BC recurrence [21] reported that the grade concordance between any two pathologists was 59–65% and overall concordance among three pathologists was 43%, with the lowest for well-differentiated and moderately differentiated tumor grades and highest for poorly differentiated. Of note, the interobserver variability in tumor grading was reported to be typical in oncology practice. As such, in 2002, the AJCC Breast Task Force did not add tumor grade to its staging criteria due to sparseness and variability of the data. Since then, the AJCC, 8th edition, has included the NGS as a recommended feature for appropriate BC staging [22]. Here, we demonstrate the impact of improved performance of Oncotype RS when combined with PDxBr in a subset of the training and validation cohorts. Applying a threshold to discriminate low versus high risk for recurrence, the Oncotype + PDxBr and Oncotype + AI image grade models were superior to Oncotype alone, suggesting that application of an improved BC grade with Oncotype RS may enhance overall risk discrimination. In context with our previous study [23], the conceptual view of using readily available materials to recapitulate genomic assays, which by nature reflect morphological attributes, warrants further investigation [24].

The TAILORx study to redistribute the intermediate-risk group continues to use clinical grade as a determinant for chemotherapy in the ≤ 50-year-old population with a recurrence score of 16–25, emphasizing the necessity to provide the most accurate grade assessment possible [25]. This study also served as the source material for the RSClin tool that identified an improvement in the prediction of distant recurrence when combining the RS score with clinical features, one of which was histologic grade [26]. Introducing an automated grade into the RSClin has the potential to further improve performance. A more recent study [27] illustrates the importance of grade variability between and within pathologists, which is more pronounced when using digital images, specifically for mitotic figure assessment and nuclear pleomorphism. Since many academic pathology departments and large commercial pathology laboratories are moving toward digital platforms for diagnosis, image-based tools that improve accuracy is increasingly important.

Several studies have highlighted histologic grade as an independent prognostic feature, specifically when genomic assays such as Oncotype DX are under consideration. A sizeable study of 1268 patients suggested that pathologic data (i.e., grade/stage) was sufficient to replace the use of the Oncotype RS for low- and high-risk individuals, reserving the assay instead for the intermediate group alone [21]. Our binomial high- and low-risk categorization for grade utilizing only the PDx AI grade addresses the current ambiguity associated with grade 2 breast cancer by redistributing 22% into the high risk category and 78% into the low risk. Anecdotally, many breast oncologists report that there is an overriding belief that grade 2 is a hybrid of grade 1 and 3 disease. Furthermore, we have also demonstrated a 99% concordance of grade 1 with AI low risk versus a redistribution of grade 3 into 44% high risk and 66% as low. The implication behind this reassignment is a potential adjustment of clinical risk which includes grade, tumor size, and stage. Additional studies are underway to evaluate. As the individual models in Fig. 3 illustrate, there is significant and incremental improvement when imaging features (representing AI-augmented BC grade) and clinical features (including age, tumor size, tumor stage, and lymph node status) are combined.

## Limitations

Although not unexpected, the number of events (14%) is low given that this cohort is classified as low risk but still important to consider due to the underlying disease potential and mortality associated with BC. Additionally, we have a limited follow-up, limited reported racial diversity and currently lack an external validation cohort. To address these deficiencies, we are actively pursuing access to several geographically diverse populations as well as completed Phase III Clinical Trial cohorts to interrogate grading system prognostication, the use of clinical risk, and alignment with genomic strategies.

## Summary and conclusion

Through advances in WSI AI-digital H&E image analysis of invasive BC, we have produced an assay to stratify patients for early-stage BC recurrence. This approach is both quantifiable and reproducible, combining MFA with standard-of-care attributes including patients’ age, tumor size, and extent of disease. By introducing novel tissue and cellular attributes such as tumor–stromal ratios and lymphocytic content, the models have produced an additional layer of biological intent for phenotyping breast cancer. Future studies with extended follow-up are in progress to facilitate treatment decision-making, enrich gene expression assays, and improve disease management within the broader breast cancer community.

## Supplementary Information

**Additional file 1.** Methods.

**Additional file 2**. **Supplementary Table 1:** PDxBr Training and Validation: Clinical Feature only model.

**Additional file 3.** **Supplementary Table 2:** PDxBr Training and Validation: Image Feature Only Model.

**Additional file 4.** **Supplementary Figure 1:** MindAct Clinical Risk Models vs. PDxBr in training (A) vs validation (B).

**Additional file 5.**. **Supplementary Figure 2:** Kaplan-Meier Comparison of Histologic Grade vs. AI-grade in Full Train and Validation Cohort.

**Additional file 6.** **Supplementary Table 3:** Demographics of Combined Training and Validation Oncotype Dataset.

**Additional file 7.** **Supplementary Table 4A-D:** Oncotype models.

**Additional file 8**. **Supplemental Figure 3:** AUC/C-index Oncotype Models.

## Data Availability

The datasets used and/or analyzed during the current study are available from the corresponding author upon reasonable request.
