# Supplementary material for: Development and validation of an AI-enabled digital breast cancer assay to predict early-stage breast cancer recurrence within 6 years
Source: Breast Cancer Res. 2022 Dec 20;24:93. doi: 10.1186/s13058-022-01592-2 (PMC9764637; doi:10.1186/s13058-022-01592-2)
Supplement: Supplementary file 1 — Additional file 1. Methods. [file 13058_2022_1592_MOESM1_ESM.docx]

**Additional Online Content**

**Development and Validation of an AI-Enabled Digital Breast Cancer Assay to Predict Early-Stage Breast Cancer Recurrence within 6 years**

Fernandez G, Prastawa M, Scott R, Marami B, Shpalensky N, Sainath Madduri A, Cascetta K, Sawyer M, Chan M, Koll G, Shtabsky A, Feliz A, Hansen T, Veremis B, Cordon-Cardo C, Zeineh J, Donovan MJ.

Additional File 1: Methods

Additional File 2: Supplementary Table 1: PDxBr Training and Validation: Clinical Feature only model

Additional File 3: Supplementary Table 2: PDxBr Training and Validation: Image Feature Only Model

Additional File 4: Supplementary Figure 1: MindAct Clinical Risk Models vs. PDxBr in training (A) vs validation (B)

Additional File 5: Supplementary Figure 2: Kaplan-Meier Comparison of Histologic Grade vs. AI-grade in Full Train and Validation Cohort

Additional File 6: Supplementary Table 3: Demographics of Combined Training and Validation Oncotype Dataset

Additional File 7: Supplementary Table 4A-D: Oncotype models

Additional File 8: Supplemental Figure 3: AUC/C-index Oncotype Models

This supplemental material has been provided by the authors to give readers additional information about their work.

**Additional File 1: Methods**

The MFA platform provides hematoxylin and eosin stain normalization, tumor region selection (invasive ductal carcinoma vs ductal carcinoma in situ), tumor volume quantitation, and tumor cell compartment characterization (epithelial vs. stroma; nuclear vs. cytoplasmic; lymphocyte compartments). The result is automated prognostic multifactorial, multi-dimensional breast cancer phenotype which encompasses some of the traditional grading elements such as tubule formation (lumen size and shape, epithelial cell organization, stromal – epithelial interface), mitotic figure detection and nuclear pleomorphism (degree of nuclear pleomorphism including shape axis-ratio, round, elliptical), nuclear envelope (smoothness, folds, irregularities), nuclear volume, and chromatin content (i.e. clumps, diffuse) but with a quantitative, advanced dynamic design that introduces cell type classification, interrelationships, tissue-geographic attributes, and inter-compartment associations (Figure 2). The derived features, which represent several thousand, were evaluated with outcome in both univariate and multivariate models. This outcome-based image feature curation and integrative filtering process represents the infrastructure of AI-grade design.

The ‘Building Blocks’ of the morphology feature array (MFA) are the relevant histologic compartments (e.g. nuclei, mitotic figures, epithelium/stroma, lymphocytes and tumor segmentation) which are identified in the image. Features derived from these Building Blocks are used to characterize these compartments and their relationships (e.g., counts, shape, density, size and topology). The features in combination with clinical parameters are the inputs to the algorithm used to calculate the probability of a patient’s risk for experiencing breast cancer recurrence defined as locoregional (ipsilateral) recurrence, distant metastasis and overall-survival. Accuracy of the Building Block is determined based on concordance of the algorithm output to the pathologist ground truth annotations - the specifics are unique for each Building Block.
