# Supplementary material for: Development and validation of an AI-enabled digital breast cancer assay to predict early-stage breast cancer recurrence within 6 years
Source: Breast Cancer Res. 2022 Dec 20;24:93. doi: 10.1186/s13058-022-01592-2 (PMC9764637; doi:10.1186/s13058-022-01592-2)
Supplement: Supplementary file 2 — Additional file 2. Supplementary Table 1: PDxBr Training and Validation: Clinical Feature only model. [file 13058_2022_1592_MOESM2_ESM.docx]

**Additional File 2: Supplementary Table 1: PDxBr Training and Validation: Clinical Features Only Model**

| Name | |  | Weight |
| --- | --- | --- | --- |
| size-ci-0.31743 | |  | -35.60 |
| ageAtDx-ci-0.37873 | |  | -32.56 |
| anatomicStageFloat-ci-0.35862 | |  | -21.94 |
| posLN-ci-0.41704 | |  | -4.58 |
| Training Model | |  |  |
| Confidence Interval | |  | 0.725 (0.698, 0.745) |
| Threshold | |  | 33.57 |
| Sensitivity | |  | 0.686 |
| Specificity |  | | 0.692 |
| PPV |  | | 0.250 |
| NPV |  | | 0.943 |
| HR |  | | 4.392 |
| p value |  | | 7.740e-25 |
| Validation Model | | |  |
| Confidence Interval |  | | 0.707 (0.658, 0.755) |
| Sensitivity |  | | 0.658 |
| Specificity |  | | 0.700 |
| PPV |  | | 0.212 |
| NPV |  | | 0.943 |
| HR |  | | 4.801 |
| p value |  | | 1.085e-09 |

Abbreviations: HR, hazard ratio; NPV, negative predictive value; PPV, positive predictive value
