# Supplementary material for: Development and validation of an AI-enabled digital breast cancer assay to predict early-stage breast cancer recurrence within 6 years
Source: Breast Cancer Res. 2022 Dec 20;24:93. doi: 10.1186/s13058-022-01592-2 (PMC9764637; doi:10.1186/s13058-022-01592-2)
Supplement: Supplementary file 3 — Additional file 3. Supplementary Table 2: PDxBr Training and Validation: Image Feature Only Model. [file 13058_2022_1592_MOESM3_ESM.docx]

**Additional File 3: Supplementary Table 2: PDxBr Training and Validation: Image Features Only Model**

| Name |  | Weight | |
| --- | --- | --- | --- |
| MitoNucleiRatioInvEpi-ci-0.37367 |  | -19.10 | |
| NucleiClusterPercentile90MSTDegree2ProportionInvEpi-ci-0.61285 |  | -13.83 | |
| NucleiSizeHighByAveInvEpi-ci-0.38705 |  | -18.6 | |
| TumorStromaRatio-ci-0.38737 |  | 9.22 | |
| Lymph_count_pertumor_count_win_15000_per10_yjreann-ci-0.62321 |  | 19.12 | |
| Tumor4_Sheetsx_x1minus_Tubulesx-ci-0.33721 |  | 1.62 | |
| Tumor4_Tubules_x1minus_Sheets-ci-0.67446 |  | 68.03 | |
| Training Model |  |  | |
| Confidence Interval |  | 0.723 (0.698, 0.745) | |
| Threshold |  | 69.50 | |
| Sensitivity |  | 0.723 | |
| Specificity |  | 0.692 | |
| PPV |  | 0.260 | |
| NPV |  | 0.943 | |
| HR |  | 3.648 | |
| p value |  | 2.170e-20 | |
| Validation Model |  | |  |
| Confidence Interval |  | 0.670 (0.634, 0.713) | |
| Sensitivity |  | 0.474 | |
| Specificity |  | 0.716 | |
| PPV |  | 0.170 | |
| NPV |  | 0.917 | |
| HR |  | 2.167 | |
| p value |  | 0.0014 | |

Abbreviations: HR, hazard ratio; NPV, negative predictive value; PPV, positive predictive value
