# Supplementary material for: Development and validation of an AI-enabled digital breast cancer assay to predict early-stage breast cancer recurrence within 6 years
Source: Breast Cancer Res. 2022 Dec 20;24:93. doi: 10.1186/s13058-022-01592-2 (PMC9764637; doi:10.1186/s13058-022-01592-2)

**Additional File 4: Supplementary Figure 1: MindAct Clinical Risk Models vs. PDxBr in training (A) vs validation (B)**


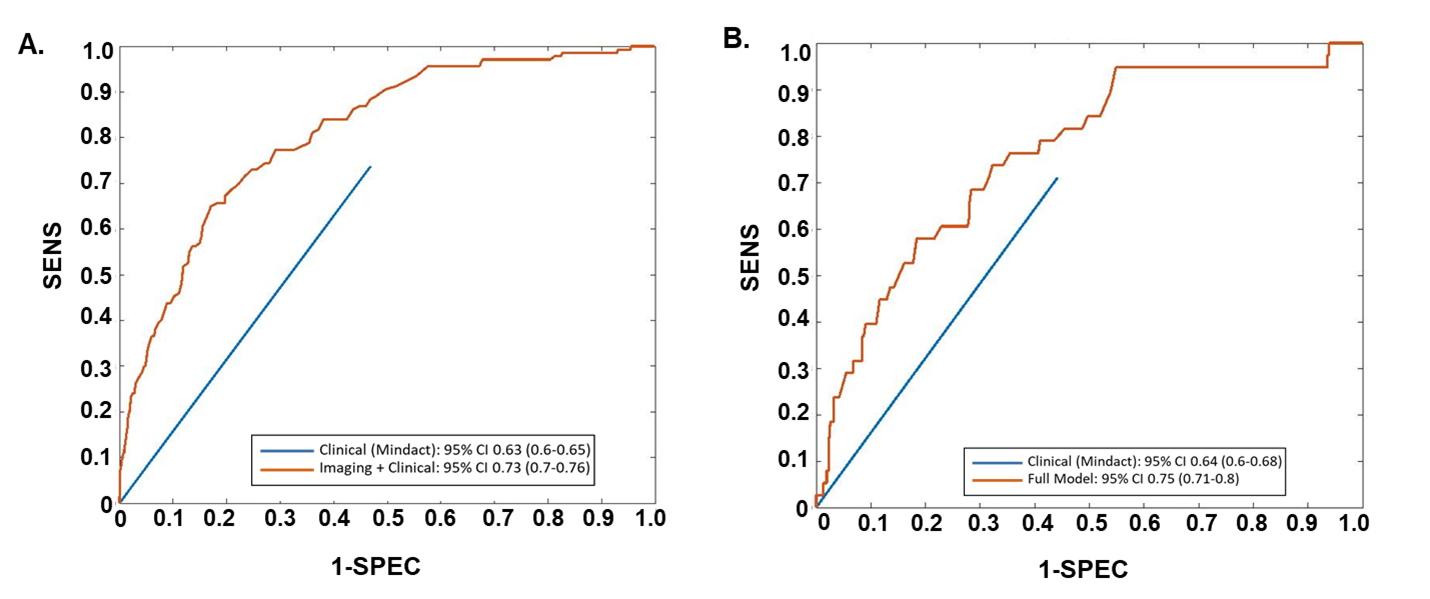

Supplement: Supplementary file 4 — Additional file 4. Supplementary Figure 1: MindAct Clinical Risk Models vs. PDxBr in training (A) vs validation (B). [file 13058_2022_1592_MOESM4_ESM.docx]
