# Supplementary material for: Development and validation of an AI-enabled digital breast cancer assay to predict early-stage breast cancer recurrence within 6 years
Source: Breast Cancer Res. 2022 Dec 20;24:93. doi: 10.1186/s13058-022-01592-2 (PMC9764637; doi:10.1186/s13058-022-01592-2)
Supplement: Supplementary file 5 — Additional file 5.. Supplementary Figure 2: Kaplan-Meier Comparison of Histologic Grade vs. AI-grade in Full Train and Validation Cohort. [file 13058_2022_1592_MOESM5_ESM.docx]

**Additional File 5: Supplementary Figure 2: Kaplan-Meier Comparison of Histologic Grade vs. AI-grade in Full Train and Validation Cohort**


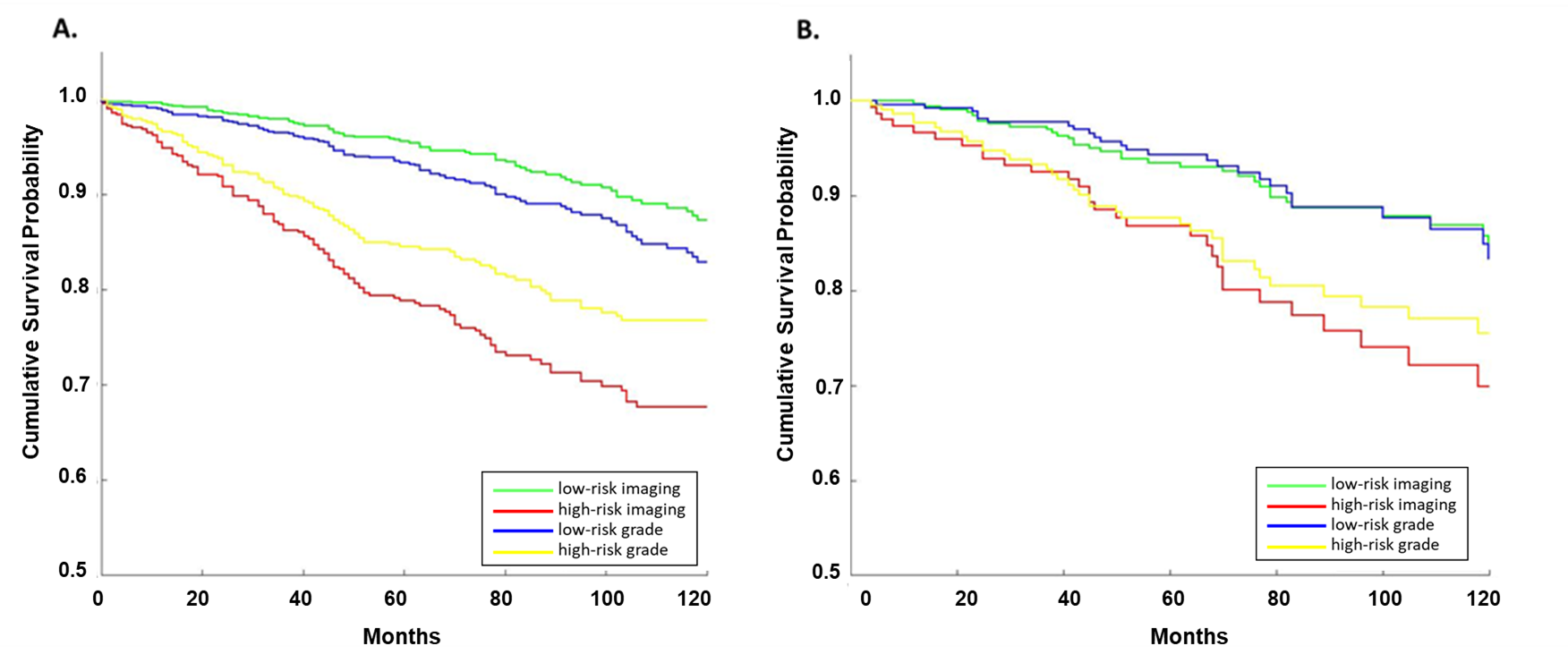


A) KM of the full training cohort comparing histologic grade and imaging; B) KM of the full validation cohort comparing histologic grade and imaging.
