# Supplementary material for: Development and validation of an AI-enabled digital breast cancer assay to predict early-stage breast cancer recurrence within 6 years
Source: Breast Cancer Res. 2022 Dec 20;24:93. doi: 10.1186/s13058-022-01592-2 (PMC9764637; doi:10.1186/s13058-022-01592-2)
Supplement: Supplementary file 6 — Additional file 6. Supplementary Table 3: Demographics of Combined Training and Validation Oncotype Dataset. [file 13058_2022_1592_MOESM6_ESM.docx]

**Additional File 6: Supplementary Table 3: Demographics of Combined Training and Validation Oncotype Dataset**

| **Combined Cohort** | **Oncotype HER2 removed** |
| --- | --- |
| **Total, N** | 599 |
| **Race/Ethnicity, N (%)** |  |
| Asian | 4 (0.7) |
| Black | 10 (2) |
| Hispanic | 3 (0.5) |
| Other | 79 (13) |
| Unknown | 162 (27) |
| White | 341 (57) |
| **Grade, N (%)** |  |
| 1 | 109 (18) |
| 2 | 332 (55) |
| 3 | 158 (26) |
| **Age at diagnosis (years)** | 57.02 [27, 57.0, 86] |
| **Tumor size (cm)** | 1.30±0.65 [0.2, 6.5] |
| **T1/2, %** | 100 |
| **Stage 1/2, %** | 100 |
| **pN0, N (%)** | 565 (95) |
| **pN1-3, N (%)** | 33 (5) |
| **ER, N (%)** |  |
| 0 | 4 (0.7) |
| 1 | 595 (99.) |
| **PR, N (%)** |  |
| 0 | 34 (6) |
| 1 | 565 (94) |
| **HER2, N (%)** |  |
| 0 | 599 (100) |
| 1 | 0 |
| **Total Events, N (%)** |  |
| 0 | 563 (94) |
| 1 | 36 (6) |
| **Time to Event (months)** | 68.00 [0.0, 63.0, 164] |
| **Chemotherapy, N (%)** |  |
| 0 | 4 (0.7) |
| 1 | 149 (25) |
| 2 (unknown) | 446 (74) |
| **Event Types, N** |  |
| LocoRegional | 22 |
| Metastasis | 7 |
| Deceased | 5 |
| Other/ second primary | 2 |

Abbreviations: ER, estrogen receptor; PR, progesterone receptor
