# Supplementary material for: Development and validation of an AI-enabled digital breast cancer assay to predict early-stage breast cancer recurrence within 6 years
Source: Breast Cancer Res. 2022 Dec 20;24:93. doi: 10.1186/s13058-022-01592-2 (PMC9764637; doi:10.1186/s13058-022-01592-2)
Supplement: Supplementary file 7 — Additional file 7. Supplementary Table 4A-D: Oncotype models. [file 13058_2022_1592_MOESM7_ESM.docx]

**Additional File 7: Supplementary Table 4A-D: Oncotype models**

**Supplemental Table 4A: Oncotype Alone**

| **Name** | **Weight** | **Cis** |
| --- | --- | --- |
| oncotypeRecurrenceScore-ci-0.35364 | 6.892 | 0.354 |
| Trainci | 0.354 | 0 |
| Threshold | 72.131 | 0 |
| Trainse | 0.375 | 0 |
| Trainsp | 0.486 | 0 |
| PPV | 0.051 | 0 |
| NPV | 0.914 | 0 |
| Hazard Ratio | 0.485 | 0 |
| Hazard Ratio (P value) | 0.041 | 0 |
| 95%CI | 0.356 | 0 |
| 95%CI Upper | 0.261 | 0 |
| 95%CI Lower | 0.447 | 0 |

Abbreviations: CI, confidence interval; NPV, negative predictive value; PPV, positive predictive value; ci, c-index; sp, specificity; se, sensitivity

**Supplemental Table 4B:**  **Oncotype + Clinical Features**

| **Name** | **Weights** | **Cis** |
| --- | --- | --- |
| OncotypeRecurrenceScore-ci-0.35364 | -1.028 | 0.354 |
| Size-ci- 0.4011 | -13.643 | 0.401 |
| AgeAtDx-ci-0.44536 | -7.804 | 0.445 |
| AnatomicStageFloat-ci-0.43951 | 0.629907 | 0.434 |
| PosLN-ci-0.43882 | -23.960 | 0.439 |
| Trainci | 0.641 | 0 |
| Threshold | 20.689 | 0 |
| Trainse | 0.625 | 0 |
| Trainsp | 0.684 | 0 |
| PPV | 0.126 | 0 |
| NPV | 0.962 | 0 |
| Hazard Ratio | 2.162 | 0 |
| Hazard Ratio (P value) | 0.021 | 0 |
| 95%CI | 0.641 | 0 |
| 95%CI Upper | 0.571 | 0 |
| 95%CI Lower | 0.714 | 0 |

Abbreviations: CI, confidence interval; NPV, negative predictive value; PPV, positive predictive value; ci, c-index; sp, specificity; se, sensitivity

**Supplemental 4C:**  **Oncotype + AI-grade**

| **Names** | **Weights** | **Cis** |
| --- | --- | --- |
| OncotypeRecurrenceScore-ci-0.35364 | -9.894 | 0.354 |
| MitoNucleiRatioInvEpi-ci-0.38193 | -3.767 | 0.382 |
| NucleiClusterPercentile90MSTDegree2ProportionInvEpi-ci-0.57835 | -4.538 | 0.578 |
| NucleiSizeHighByAveInvEpi-ci-0.36519 | -3.826 | 0.365 |
| TumorStromaRatio-ci-0.30993 | 14.819 | 0.319 |
| Lymph_count_per_tumor_count_win_15000_per10_yj_reann-ci-0.68528 | 17.519 | 0.685 |
| Tumor4_Sheetsx_x1minus_Tubulesx-ci-0.31254 | -1.617 | 0.313 |
| Tumor4_Tubules_x1minus_Sheets-ci-0.68637 | 8.334 | 0.686 |
| Trainci | 0.728 | 0 |
| Threshold | 54.894 | 0 |
| Trainse | 0.750 | 0 |
| Trainsp | 0.751 | 0 |
| PPV | 0.180 | 0 |
| NPV | 0.976 | 0 |
| Hazard Ratio | 4.603 | 0 |
| Hazard Ratio (P value) | 8.14E-06 | 0 |
| 95%CI | 0.723 | 0 |
| 95%CI Upper | 0.668 | 0 |
| 95%CI Lower | 0.787 | 0 |

Abbreviations: CI, confidence interval; NPV, negative predictive value; PPV, positive predictive value; ci, c-index; sp, specificity; se, sensitivity

**Supplemental 4D: Onco + PDxBr Model**

| **Names** | **Weights** | **Cis** |
| --- | --- | --- |
| OncotypeRecurrenceScore-ci-0.35364 | -13.108 | 0.354 |
| MitoNucleiRatioInvEpi-ci-0.38193 | 2.563 | 0.3819 |
| NucleiClusterPercentile90MSTDegree2ProportionInvEpi-ci-0.57835 | -14.955 | 0.579 |
| NucleiSizeHighByAveInvEpi-ci-0.36519 | -4.559 | 0.365 |
| TumorStromaRatio-ci-0.30993 | -10.816 | 0.310 |
| Lymph_count_per_tumor_count_win_15000_per10_yj_reann-ci-0.68528 | 17.159 | 0.685 |
| Tumor4_Sheetsx_x1minus_Tubulesx-ci-0.31254 | 3.497 | 0.313 |
| Tumor4_Tubules_x1minus_Sheets-ci-0.68637 | 12.607 | 0.686 |
| AgeAtDx-ci-0.44536 | -9.790 | 0.445 |
| AnatomicStageFloat-ci-0.43951 | -8.215 | 0.440 |
| PosLN-ci-0.43882 | -22.872 | 0.439 |
| Age2Size2-ci-0.40095 | -1.483 | 0.401 |
| Trainci | 0.764 | 0 |
| Threshold | 43.290 | 0 |
| Trainse | 0.667 | 0 |
| Trainsp | 0.805 | 0 |
| PPV | 0.200 | 0 |
| NPV | 0.971 | 0 |
| Hazard Ratio | 6.134 | 0 |
| Hazard Ratio (P value) | 1.19E-07 | 0 |
| 95%CI | 0.760 | 0 |
| 95%CI Upper | 0.699 | 0 |
| 95%CI Lower | 0.820 | 0 |

Abbreviations: CI, confidence interval; NPV, negative predictive value; PPV, positive predictive value; ci, c-index; sp, specificity; se, sensitivity
