# Supplementary material for: Development and validation of an AI-enabled digital breast cancer assay to predict early-stage breast cancer recurrence within 6 years
Source: Breast Cancer Res. 2022 Dec 20;24:93. doi: 10.1186/s13058-022-01592-2 (PMC9764637; doi:10.1186/s13058-022-01592-2)
Supplement: Supplementary file 8 — Additional file 8. Supplemental Figure 3: AUC/C-index Oncotype Models. [file 13058_2022_1592_MOESM8_ESM.docx]

**Additional File 8: Supplemental Figure 3: AUC/C-index Oncotype Models**

**
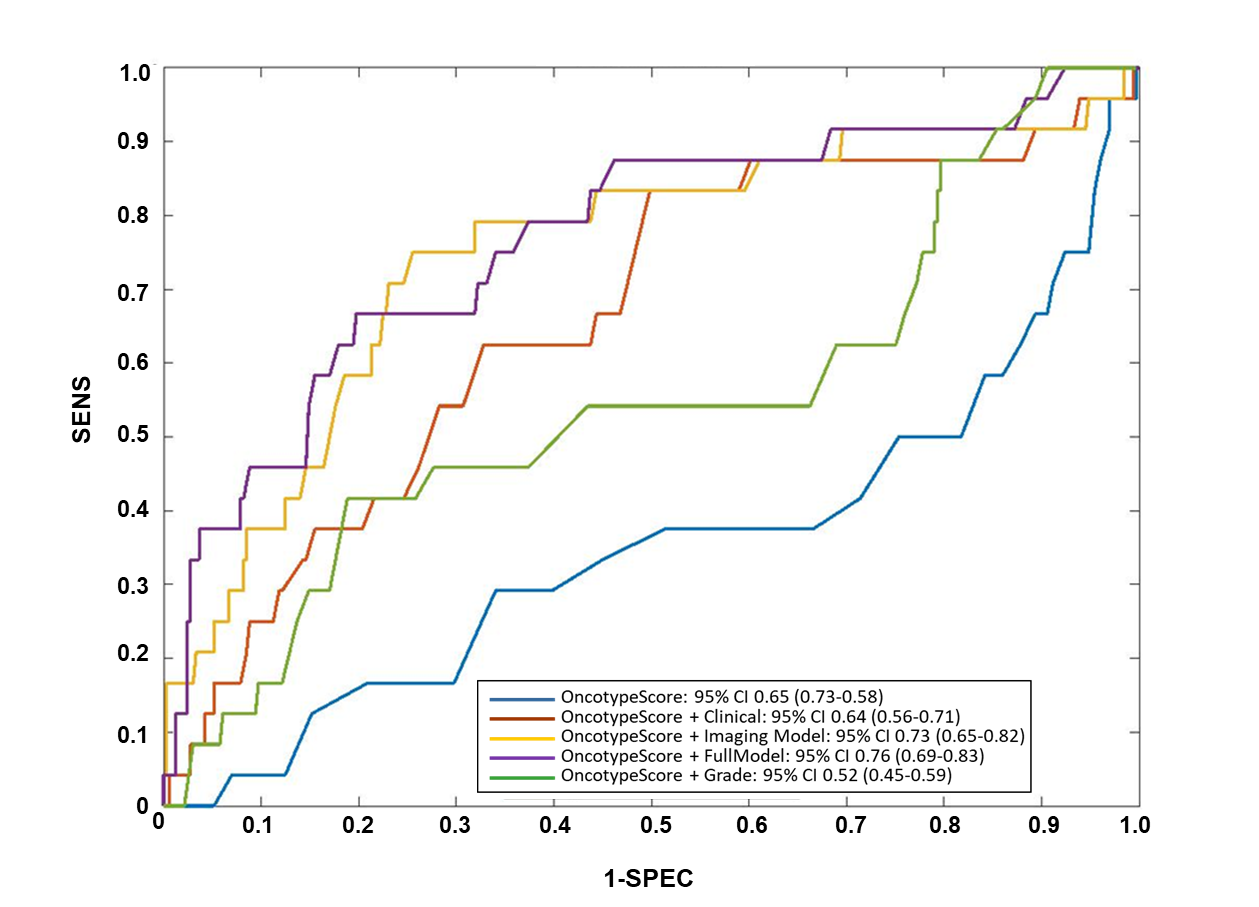
**

AUC curves of the Oncotype Subgroup with all sub-models represented demonstrating incremental improvement when adding AI-grade and the full PDxBr model. Note: AUC values < 0.5 are displayed as the reciprocal value, i.e. Oncotype RS actual AUC is 0.35 and displayed on the ROC curve as 0.65.
